# Systematic analysis of coronary artery disease datasets revealed the potential biomarker and treatment target

**DOI:** 10.18632/oncotarget.17426

**Published:** 2017-04-26

**Authors:** Yan Shi, Sijin Yang, Man Luo, Wei-Dong Zhang, Zun-Ping Ke

**Affiliations:** ^1^ Department of Emergency, The Affiliated Huai′an Hospital of Xuzhou Medical University and The Second People′s Hospital of Huai′an, Huai′an, China; ^2^ Department of Heart Encephalopathy, Affiliated Traditional Chinese Medicine Hospital, Southwest Medical University, Luzhou, China; ^3^ Department of Emergency, Huai′an First People′s Hospital, Nanjing Medical University, Huai′an, China; ^4^ Department of Cardiology, People's Hospital of Xuyi, Jiangsu, Xuyi, China; ^5^ Department of Cardiology, The Fifth People′s Hospital of Shanghai, Fudan University, Shanghai, China

**Keywords:** coronary artery disease, systematic analysis, microarray, gene expression omnibus (GEO), neutrophil degranulation

## Abstract

Coronary artery disease caused about 1 of every 7 deaths in the United States and early prevention was potential to decrease the incidence and mortality. We aimed to figure the genes involving in the coronary artery disease using meta-anlaysis. Five datasets of coronary heart disease from GEO series were retrieved and data preprocessing and quality control were carried out. Moderated t-test was used to decide the differentially expressed genes for a single dataset. And the combined p-value using systematic-analysis methods were conducted using MetaDE. The pathway enrichment was carried out using Reactome database. Protein-protein interactions of the identified differentially expressed genes were also analyzed using STRING v10.0 online tool. After removing unidentified or intermediate samples and a total of 238 cases and 189 matched or partially matched control from five microarray datasets were retrieved from GEO. Six different quality control measures were calculated and PCA biplots were plotted in order to visualize the quantitative measure. The first two PCs captured 91% of the variance and we decided to include all of the datasets for systematic analysis. Using the FDR cut-off as 0.1, nine genes, including LFNG, ID3, PLA2G7, FOLR3, PADI4, ARG1, IL1R2, NFIL3 and MGAM, were differentially expressed according to maxP. Their protein-protein interactions showed that they were closely connected and 24 Reactome pathways were related to coronary artery disease. We concluded that pathways related to immune responses, especially neutrophil degranulation, were associated with coronary heart disease.

## INTRODUCTION

Coronary artery disease caused about 1 of every 7 deaths, which corresponded to 375295 deaths, in the United States in 2011 [1]. It was estimated that about 635,000 and 300,000, respectively, Americans have a new or recurrent coronary attach every year. Death rates of coronary heart disease have fallen from 1968 to the present and about 47% of the decrease in deaths caused by coronary artery disease contributed to treatments, including secondary preventive therapies after myocardial infarction or revascularization, initial treatments for acute myocardial infarction or unstable angina and so forth [2]. It suggested that early prevention of coronary heart disease was effective to some extent.

Microarray analysis has been used as a practical approach to study gene expression changes, which may help the early diagnosis of coronary heart disease [3]. Despite their great promise, a lot of studies have reported that findings pf microarray data were not reproducible or were sensitive to the data perturbations [4, 5]. Even worse, microarray used over 10 thousand probes on tens or hundreds of samples, which exacerbated the accuracy of the potential predictors.

As a result, the systematic analysis was used to increase the reliability and generalizability of results. Through systematic analysis, we aimed to obtain a more precise set of differentially expressed genes, and analyzed their biological functions. In this study, we utilized the five public microarray datasets from Gene Expression Omnibus (GEO) repository [6] to figure out the genes which were differentially expressed in patients with coronary heart disease and control using combined p values and try to give suggestions on the biomarkers for the early prevention and treatment according to the functions of these genes.

## RESULTS

### Quality of datasets

**Figure 1:**
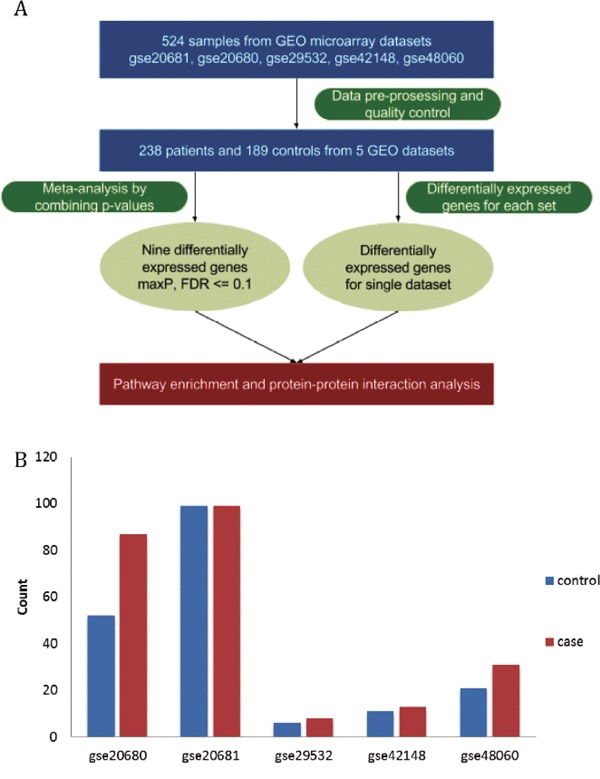
Overview of the systematic analysis and datasets of the coronary heart disease **(A)** The workflow of this study. **(B)** The number of cases (red) and controls (blue) in the five datasets of the coronary heart disease.

Five microarray datasets of patient samples with coronary heart diseases for which matched clinical information was available were obtained from GEO by using GEOquery (Table 1). After removing unidentified or intermediate samples and a total of 238 cases and 189 matched or partially matched control were selected for further analysis (Figure 1A). The detailed information of these five microarray datasets was summarized in Table 1 and Figure 1B.

**Table 1:** The GEO datasets used in this study

GEO Accession	Platform	Source DOI	control	case
gse20681	GPL4133	10.1186/1755-8794-5-58	99	99
gse20680	GPL4133	10.1186/1755-8794-4-26	52	87
gse29532	GPL5175	10.1016/j.cca.2013.03.011	6	8
gse42148	GPL13607	NA	11	13
gse48060	GPL570	10.1016/j.yjmcc.2014.04.017	21	31

**Figure 2:**
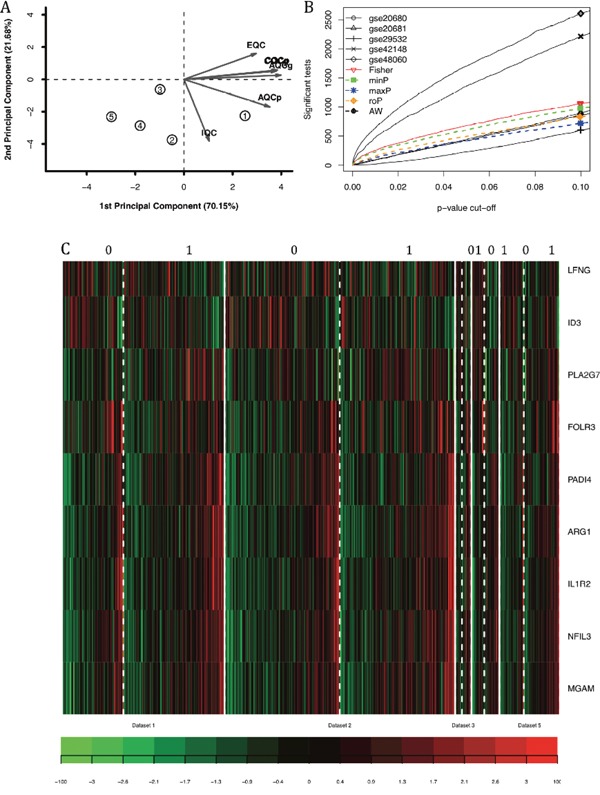
The systematic analysis of differentially expressed genes between patients with coronary heart diseases and controls by combining p-value **(A)** PCA biplot of six quality control measures in five datasets. **(B)** The number of differentially expressed genes plotted as FDR in the analysis of five different datasets. **(C)** The heatmap identifying the differentially expressed gene in cases and controls subjected to maxP systematic analysis when FDR was lower than 0.1.

Six quality control measures were calculated (Table 2) and PCA biplots (Figure 2A) were plotted in order to visualize the quantitative measure. The first two PCs also captured a high percentage of variance (91%), and the studies were more scattered in the plot. For example, GSE20680 had better scores in IQC, CQCp and AQCp while GSE20681 had better performance in CQCg, CQCp, AQCg and AQCp. GSE20681 and GSE48060 had relatively poor performance. One concern is that the EQC were poor in all five datasets, which suggested that the genes involved in coronary heart disease might not be finely defined in Biocarta pathways. Considering the PCA biplots, the six quality control criteria and the limited datasets, we decided to include all of the datasets for systematic analysis.

**Table 2:** Quantitative quality control measures of coronary heart disease studies

Dataset	Study	IQC	EQC	CQCg	CQCp	AQCg	AQCp	Rank
1	gse20680	6.25	0.9	1.84	8.58	1.7	9.27	2.5
2	gse20681	4.5	1.1	10.91	27.02	4.29	10.78	1.5
3	gse29532	2.85	0.53	0.24	2.42	0.08	0.01	4.5
4	gse42148	0.61	1.1	2.39	9.34	1.6	2.73	2.83
5	gse48060	4.91	0.91	0.24	0.84	0.8	3.73	3.67

### Differentially expressed genes

Five main systematic analysis methods by combining p-value in MetaDE package were carried out including maxP, minP, roP, AW and Fisher. The counts of differentially expressed genes by each independent datasets and by selected combined p-value were listed in Table 3. Totally seven differentially expressed genes were detected by maxP and roP evaluation criteria, respectively, using detection competency curves and false discovery rate (FDR) cut-off less than 0.05. If the FDR cut-off was set as 0.1, 9 genes were differentially expressed (Figure 2C).

**Table 3:** The number of differentially expressed genes in the five datasets of coronary heart disease using moderated-t test and meta-analysis combined p value

Cutoff	gse20681	gse20680	gse29532	gse42148	gse48060	roP	maxP
p <= 0.01	70	89	17	506	633	163	136
p <= 0.05	415	418	227	1445	1697	500	423
FDR <= 0.01	0	0	0	1	3	2	2
FDR <= 0.05	0	2	0	9	109	7	7

These nine genes had different patterns across the samples. For example, LFNG and ID3 were highly expressed in control samples, while ARG1 and IL1R2 were highly expressed in patients with coronary heart disease. Such expression patterns matched their functions which were reviewed in detail in the discussion section. The number of differentially expressed genes were plotted as a function of false discovery rate FDR in the analysis of five different datasets and the five different systematic analysis algorithms (Figure 2B). And it showed that GSE48060 and GSE42148 performed the best.

### Function analysis

**Figure 3:**
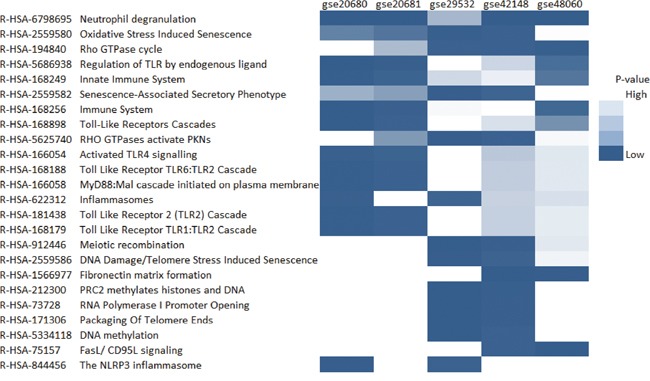
The heatmap of enriched pathways from Reactome Only the pathways which were significantly enriched with the cutoff of FDR lower than 0.05 in at least two datasets were shown in the plot.

The pathways shared by at least three datasets were plotted as heatmap (Figure 3) with a cutoff of FDR lower than 0.05 using Reactome database [15]. We identified 24 pathways related to coronary artery disease by these criteria. Notably, most of these pathways were associated with the immune system. Neutrophil degranulation seemed to be the most important pathways associated with coronary heart disease.

### Protein-protein interactions

**Figure 4:**
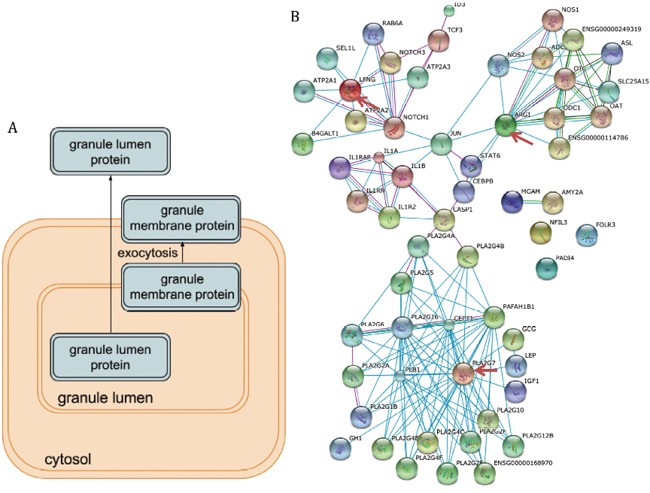
The function analysis of the differentially expressed genes **(A)** The pathway illustration of neutrophil degranulation which was the top enriched pathway. **(B)** The protein-protein interactions between the nine differentially expressed genes and their interactors. The important hubs (LFNG, ARG1, and PLA2G7) were annotated by the red arrows.

The protein-protein interactions of the identified differentially expressed genes showed that they were closely connected and played a central role *in vivo* (Figure 4B). For example, ARG1, LFNG, and PLA2G7 are hubs for this network which suggested their important function in human body.

## DISCUSSION

### Differentially expressed genes in patients with coronary heart disease

With the microarray expression data, we can figure out the genes whose expression levels help diagnose the diseases or identify the most suitable treatment. However, microarray data sometimes were not reproducible or were too sensitive to the mildest data perturbations [3]. Moreover, over ten thousand probes are investigated in only tens or hundreds of biological samples, which increase the false positive targets dramatically. As a result, it is a good idea to utilize different datasets of similar experiment designs to decrease the false positives.

In this study, we combined five datasets of coronary heart disease and used the combined maxP value to figure out nine important and reproducible genes which were differentially expressed between patients and controls. These nine genes were LFNG, ID3, PLA2G7, FOLR3, PADI4, ARG1, IL1R2, NFIL3 and MGAM. These genes were differentially expressed in all five microarray datasets, which suggested that their significant roles in the coronary heart disease. When the protein-protein interactions of these nine genes were investigated, we found that there genes had many interactors (Figure 4B), which indicated that their critical roles *in vivo*. Notably, LFNG, PLA2G7, and ARG1, which were annotated by the red arrows, were obviously hubs in the protein-protein interaction networks.

LFNG (lunatic fringe) is a member of the fringe gene family and acts in the Notch receptor pathway, which regulated macrophage activation and cardiovascular calcification [7]. Mutations on Notch1 pathways led to high rate of coronary heart disorders like stenosis and calcification. ID3 (inhibitor of DNA binding-3) was shown to be an upstream regulator to protects against the formation of atherosclerosis and the SNP in the coding region of the ID3 gene were directly associated with coronary artery pathology [8]. Such findings matched our results that LFNG and ID3 were lowly expressed in patients with coronary heart diseases, whose low expression levels might contribute to the disease progression. ARG1 regulated the C-reactive protein (CRP) levels whose high levels were risk factors of coronary heart disease [9]. The mutations on PLA2G7 gene have been reported to be associated with coronary artery disease since 15 years ago and some alleles had been proved to increase the risk of coronary heart disease [10]. Again, these findings supported our results that these genes were highly expressed in patients. Other five genes were also closely related with coronary heart disease progression or prognosis in many studies [11–13].

### Neutrophil degranulation and coronary heart disease

We identified 24 pathways related to coronary artery disease. Notably, most of these pathways were associated with immune system. It has been reported that innate and adaptive immune responses played critical roles in the development and progression of coronary heart disease [14]. The primary cause of coronary artery disease, atherosclerosis was widely accepted as a chronic inflammatory disease. It supported our findings.

Neutrophil degranulation was the top one pathway associated with coronary heart disease in our list, which was served as a case study. Neutrophils are important inflammatory cells and bone from marrow-derived white blood cells. Then they migrate from the bloodstream to sites of tissue inflammation and induce inflammation by undergoing burst and degranulation which was illustrated in Figure 4A [15]. The immune function of neutrophils determined the degranulation will alleviate the coronary heart disease. For example, it was noticed that neutrophils degranulation was able to mediate the damage of the vascular and myocardial [16]. Moreover, the function of stimulated neutrophils secreted proteolytic neutral proteases which in further promoted the detachment of endothelial cells from vessel walls and the adherence of platelets to subendothelial collagen and fibronectin [17]. Ricevuti, Mazzone [18] found that neutrophil aggregation and oxygen metabolites release increased in the coronary sinuses of patients with coronary heart disease. Although there were a lot of studies about the association between neutrophils and coronary heart disease, the attention decreased dramatically in the recent 10 years. We proposed that neutrophils may function critically in the progression of coronary heart disease and serve as the treatment targets.

In conclusion, we aggregated five coronary heart disease microarray datasets from GEO series by systematic analysis. Nine genes which were significantly differentially expressed in all the five datasets were identified. These genes played important roles in patients with coronary artery disease according to the expression levels, protein-protein interactions and enriched pathways. We also concluded that pathways related to immune responses were enriched with the differentially expressed genes and neutrophil degranulation was one of the most important processes.

## MATERIALS AND METHODS

### Datasets

Coronary heart disease and myocardial infarction were used as the keyword to search in GEO series (https://www.ncbi.nlm.nih.gov/geo/browse/?view=series). After removing datasets which were not obtained from patients’ tissues or were short of proper controls, 5 datasets were used to study the expression profiles in patients with coronary heart diseases. The processed data were downloaded as the series matrix using R package GEOquery [19]. The mRNA expression levels of targeted patients and controls were extracted from all the samples and were transformed into log2 scale before further analysis. In GSE20681, 99 cases and 99 controls were analyzed by expression profiling microarray using platform GPL4133 [20]. In GSE20680, 52 control and 87 cases were analyzed using the same platform, after intermediates cases were removed [21]. Datasets GSE29532 contained totally 55 samples at different time points, and only the expression profiles on patient admission were extracted in this study, which contained only 6 controls and 8 cases using platform GPL5175 [22]. GSE4128 analyzed 11 controls and 13 patients in Asian Indians using platform GPL13607. In GSE48060, 21 control and 31 myocardial infarction patient groups were analyzed under platform GPL570 [23].

### Data pre-processing

The expression levels of all datasets were transformed into log2 scale. R package MetaQC were utilized to finish the preprocessing of datasets [24]. The largest interquartile range (IQR) of expression values were used to represent the genes with multiple probe IDs. The expression levels of 5 datasets were first merged together according to the gene symbol and the genes that appeared in less than 4 datasets were filtered out. Given the fact that most genes were not expressed or not informative *in vivo*, 20% of unexpressed genes and 20% of uninformative genes were removed in order to decrease false positive.

### Quality control

Quantitative quality control measures were calculated to represent the quality of these datasets with the help of MetaQC [24]. The measures included internal quality control index (IQC), external quality control index (EQC), accuracy quality control index for genes or pathways (AQCg and AQCp) and consistency of differential expression quality control (CQCg and CQCp) indexes. IQC represented the internal homogeneity of co-expression, which identified potentially inconsistent or outlier studies from quantified co-expression dissimilarity. EQC index was calculated with the supervision of external pathway database MSigDB. The Biocarta and all pathways of version 5.2 from MSigDB were applied to evaluate its consistency with each study. AQC and CQC aimed at quantifying the reproducibility of differentially expressed genes or pathways detected in an individual study compared to those detected by systematic analysis from all other studies.

Principal component analysis (PCA) biplots were plotted with the help of MetaQC to visualize the quality of studies in systematic analysis. The six quality control measures were projected into a 2D space; that is, the coordinates of each quality criterion were determined by its correlation to the first two principal coordinates.

### Identification of differentially expressed genes

MetaDE package provides functions for conducting 5 different systematic analysis methods for differential expression analysis [25]; that is, Fisher, adaptively weighted Fisher (AW), minimum p-value (minP), maximum p-value (maxP) and r^th^-ordered p-value (roP). Moderated t-test was used to decide the differentially expressed genes for a single dataset. The heatmap of the differentially expressed genes under 0.1 FDR threshold across studies where created. To assess the performance of these different methods, we compared the numbers of detected differentially expressed genes from different methods under different p-value thresholds using detection competency curves.

### Function analysis

The pathway enrichment was carried out using Reactome database and FDR adjustment was applied to identify significantly enriched pathways [26, 27]. The pathways shared by at least three datasets were plotted as heatmap. Protein-protein interactions of the identified differentially expressed genes were also analyzed using STRING v10.0 online tool that visualizes known and predicted protein-protein interactions [28].
